# Appropriate homoplasy metrics in linked SSRs to predict an underestimation of demographic expansion times

**DOI:** 10.1186/s12862-017-1046-4

**Published:** 2017-09-11

**Authors:** Diego Ortega-Del Vecchyo, Daniel Piñero, Lev Jardón-Barbolla, Joost van Heerwaarden

**Affiliations:** 1 Departamento de Ecologia Evolutiva, Instituto de Ecologia, Universidad Nacional Autónoma de México, Mexico City, Mexico; 2 Department of Integrative Biology, University of California, Berkeley, USA; 3 Plant Production Systems, Wageningen University, Wageningen, the Netherlands; 4 Centro de Investigaciones Interdisciplinarias en Ciencias y Humanidades, Universidad Nacional Autónoma de México, Mexico City, Mexico

**Keywords:** Homoplasy, Haplotypes, SSRs, Demography

## Abstract

**Background:**

Homoplasy affects demographic inference estimates. This effect has been recognized and corrective methods have been developed. However, no studies so far have defined what homoplasy metrics best describe the effects on demographic inference, or have attempted to estimate such metrics in real data. Here we study how homoplasy in chloroplast microsatellites (*cpSSR*) affects inference of population expansion time. *cpSSRs* are popular markers for inferring historical demography in plants due to their high mutation rate and limited recombination.

**Results:**

In *cpSSRs*, homoplasy is usually quantified as the probability that two markers or haplotypes that are identical by state are not identical by descent (Homoplasy index, *P*). Here we propose a new measure of multi-locus homoplasy in linked *SSR* called Distance Homoplasy (*DH*), which measures the proportion of pairwise differences not observed due to homoplasy, and we compare it to *P* and its per *cpSSR* locus average, which we call Mean Size Homoplasy (*MSH)*. We use simulations and analytical derivations to show that, out of the three homoplasy metrics analyzed, *MSH* and *DH* are more correlated to changes in the population expansion time and to the underestimation of that demographic parameter using *cpSSR*. We perform simulations to show that Approximate Bayesian Computation (*ABC*) can be used to obtain reasonable estimates of *MSH* and *DH*. Finally, we use *ABC* to estimate the expansion time, *MSH* and *DH* from a chloroplast *SSR* dataset in *Pinus caribaea.* To our knowledge, this is the first time that homoplasy has been estimated in population genetic data.

**Conclusions:**

We show that *MSH* and *DH* should be used to quantify how homoplasy affects estimates of population expansion time. We also demonstrate how *ABC* provides a methodology to estimate homoplasy in population genetic data.

**Electronic supplementary material:**

The online version of this article (doi:10.1186/s12862-017-1046-4) contains supplementary material, which is available to authorized users.

## Background

The study of historical demography is important for understanding the ecology and evolution of species. In particular, timing population size changes allows the discussion of past population patterns in the context of historical geological events such as island formation [1] and climate change [2]. One popular source of information to infer past population dynamics is the genealogical signal contained in linked polymorphic markers [3, 4], such as chloroplast microsatellites (*cpSSRs*). As highlighted in recent reviews [5, 6], *cpSSRs* are widely used in plant studies. *cpSSRs* remain popular despite the ascent of genome wide sequencing tools such as Restriction site-associated *DNA* sequencing (*RADseq*) [7], Genotyping-by-sequencing (*GBS*) [8] and targeted sequencing [9] due to two appealing properties: 1) their high mutation rate, ranging from 10^−6^ to 10^−2^ mutations per locus per generation [10], and 2) they can be applied in plant non-model species where few genomic resources have been developed [11].

High mutation rates combined with an approximately step-wise transition between allelic states make *cpSSRs* prone to homoplasious mutations. Homoplasy takes place in a *cpSSR* locus when different alleles at the locus are identical by state but are not identical by descent [12]. Two *cpSSRs* copies of a locus are defined to be identical by state when they have the same size and are defined as identical by descent when there has not been a mutation since their divergence from a common ancestor. Previous studies have quantified the fraction of the homoplasy, called Molecularly accessible size homoplasy (*MASH*) [12–14] by measuring the differences in the *DNA* sequence of *SSRs* of identical size. Although that approach can reveal a fraction of the homoplasy in *SSRs*, it ignores the homoplastic events due to polymorphisms that lead to *DNA* sequences identical by state but not identical by descent. Therefore, *MASH* does not provide a direct estimate of homoplasy.

The occurrence of homoplasy is an important limitation of *cpSSR* based demographic inference in scenarios of population expansions, causing decreased ability to detect population growth [15] and to systematic underestimation of the expansion time [16]. Although some pseudo-likelihood and Bayesian methods of demographic inference [16, 17] successfully correct for homoplasy*,* they provide little insight into the relationship between homoplasy and the estimation of demographic parameters, nor do they provide estimates of homoplasy itself. In fact, to our knowledge, no formal analysis of the quantitative relation between homoplasy and the underestimation of the expansion time exists to date. Part of the reason is that the concept of homoplasy was developed to describe the proportion of haplotypes or markers that are identical by descent compared to those that are identical by state, while the problem of erroneous demographic inference is linked to an underestimation of the number of mutations between lineages. To illustrate this, the most common measure of homoplasy, the homoplasy index *P* [12], describes the probability that two *cpSSR* identical by state are not identical by descent. In the case of haplotypes composed of linked *cpSSR*, *P* has been defined as the probability that two haplotypes identical by state are not identical by descent and is dependent on the multi-locus heterozygosity [15]. This is the definition of *P* we will employ here. While simulation studies show that higher values of *P* are associated with an underestimation of the expansion time [15], other studies have found that multi-locus heterozygosity is not particularly sensitive to homoplasy [18], suggesting that *P* may not be the most appropriate measure for describing effects on demographic inference. This motivates the necessity to propose alternative measures of homoplasy that are more directly relevant to demographic inference and that would allow for meaningful quantifications of the effects of homoplasious mutations on the estimation of the expansion time.

In this paper, we propose a new homoplasy metric. We analyze the relationship between three homoplasy metrics, including our proposed metric, and the underestimation of the expansion time. Second, we evaluate the extent to which these homoplasy metrics can be estimated from simulated *cpSSR* data using Approximate Bayesian Computation (*ABC*). Finally, we quantify the level of homoplasy in a real dataset from *Pinus caribaea*, providing an empirical estimate of homoplasy from population genetic data.

## Methods

### Dataset simulations under a stepwise demographic expansion model

Throughout this study we assume a stepwise demographic expansion model [3]. The model consists on three parameters: *θ*_0_ = 2*LN*_0_ *u*, *θ*_1_ = 2*LN*_1_ *u* and *τ* = 2*Ltu*, where *u* is the mutation rate per generation at each linked *SSR*, *N*_0_ and *N*_1_ are the effective population sizes before and after the expansion, *L* is the number of linked *SSR* loci and *t* is the time in generations since the expansion.

We generated two sets of haplotypes, *hISM* and *hSMM*, in the coalescent simulations under the stepwise demographic expansion model used in this study. We used the same genealogy along with the set of mutations falling in each branch of the genealogy to generate *hISM* and *hSMM* from each coalescent simulation. *hISM* represents a set of linked multi-locus *SSR* haplotypes evolving under the infinite sites model, *ISM* [19] while *hSMM* are a set of linked multi-locus *SSR* haplotypes that evolved under the symmetrical stepwise mutation model, *SMM* [20]. The haplotypes *hISM* are free of homoplasy while the haplotypes *hSMM* can contain homoplasious mutations. These coalescent simulations were performed using a modified a version of the coalescent simulator *msHOT* [21, 22]. The modified version of *msHOT* is available at https://github.com/dortegadelv/HomoplasyMetrics.

### Measures of homoplasy

We studied the relationship between homoplasy and the underestimation of the population expansion time τ using three different measures.

The first metric is the commonly used *homoplasy index* (*P*) [12] as used by [15]:

1 $$ P=1-\frac{1-{H}_{ISM}}{1-{H}_{SMM}}=1-\frac{F_{ISM}}{F_{SMM}} $$

Where *H*_*ISM*_and *H*_*SMM*_ are the expected heterozygosities [23] per haplotype estimated in a set of haplotypes containing *L* linked loci evolving under the infinite sites model (*hISM)* and the stepwise mutation model (*hSMM*), respectively. *F*_*ISM*_ and *F*_*SMM*_ are the expected homozygosities in the set of haplotypes *hISM* and *hSMM*. Note that *F*_*SMM*_ is directly observable from the data, while *F*_*ISM*_ is not, in real data of a set of haplotypes *hSMM*.

We also use the per *SSR* locus average of *P*, which we call Mean Size Homoplasy (*MSH*). It estimates the mean reduction in heterozygosity per *SSR* locus. This can be interpreted as the mean homoplasy index *P* per individual loci. It can be expressed as:

2 $$ MSH=1-\frac{\sum_{i=1}^L\frac{1-{H}_{ISM}^i}{1-{H}_{SMM}^i}}{L}=1-\frac{\sum_{i=1}^L\frac{F_{ISM}^i}{F_{SMM}^i}}{L} $$

Where *L* is the number of *SSR* in the haplotype. ${H}_{ISM}^i$ and ${H}_{SMM}^i$ are the expected heterozygosities at the *i* locus in *hISM* and *hSMM,* respectively*.* ${F}_{ISM}^i$ and ${F}_{SMM}^i$ are the expected homozygosities at the *i* locus in *hISM* and *hSMM.*

Inference of demographic growth using haplotypes with linked microsatellites is typically based on the distribution of pairwise differences between multi-locus haplotypes, also known as the mismatch distribution, as the shape of this distribution is determined by the time and magnitude of historical population expansions [4]. Based on this, here we present a new metric, distance homoplasy (*DH*), which quantifies the proportion of mutations separating two multi-locus haplotypes that are not observed due to homoplasy. Our rationale for using this measure are studies that use the mode of the distribution of pairwise differences as the basis for estimating τ [4]. Therefore, underestimation of the proportion of pairwise differences should impact the mismatch distribution which in turn should alter the inference of τ. *DH* is expressed as:

3 $$ DH=\frac{\pi_{ISM}-{\pi}_{SMM}}{\pi_{ISM}} $$

Where *π*_*SMM*_ and *π*_*ISM*_ are the mean number of differences between two haplotypes using the haplotypes *hSMM* and *hISM*, respectively.

### Expected values of π, F^i^ and F in a stepwise demographic expansion model

We derived the expected values for the diversity statistics *π*, *F* ^*i*^ and *F* as a function of the mutation rate *u* of each linked *SSR*, the number of linked simulated *SSR’s L* and the coalescent time *T*_*ij*_, in number of generations, between a pair of haplotypes *i* and *j* present in the sample. We use the following equation $E\left[\lambda \right]=E\left[E\left[\lambda |{T}_{ij}\right]\right]={\sum}_{x=1}^tE\left[\lambda |{T}_{ij}=x\right]P\left({T}_{ij}=x\right)$ where *λ* stands for any diversity statistic and *t* is the time in generations since the expansion. *T*_*ij*_ is scaled in units of *N* generations. We explain how to obtain the values of *E*[*λ*| *T*_*ij*_] for every diversity statistic under the *ISM* and *SMM* in the Appendix. The probability distribution of *T*_*ij*_ under a stepwise demographic expansion model is equal to:

4 $$ P\left({T}_{ij}=x\right)=\left\{\begin{array}{cc}\raisebox{1ex}{$1$}\!\left/ \!\raisebox{-1ex}{$N$}\right.\left({e}^{-\raisebox{1ex}{$x$}\!\left/ \!\raisebox{-1ex}{$N$}\right.}\right)& \kern1.25em 0\le x<t-1\\ {}1-{\sum}_{x=1}^{t-1}P\left({T}_{ij}=x\right)& \kern1.5em x=t\end{array}\right. $$

Where *N* is the effective population time in the present. The probability distribution of *T*_*ij*_ is divided into two phases: 1) After the expansion, the population keeps a constant population size and, therefore, $$ P\left({T}_{ij}=x\right)=\raisebox{1ex}{$1$}\!\left/ \!\raisebox{-1ex}{$N$}\right.{e}^{-\raisebox{1ex}{$x$}\!\left/ \!\raisebox{-1ex}{$N$}\right.} $$ during that period of time. 2) Before the expansion, all individuals must coalesce quickly at a time very close to the expansion time *T*_*ij*_ = *t* assuming that the population size is very small. To model that effect, we assume that all individuals coalesce exactly at time *T*_*ij*_ = *t* if they have not already coalesced going forward in time.

The equations shown above to estimate the expected value of the diversity statistics are used to obtain estimates of the homoplasy parameters *P, MSH* and *DH*. As an example, following equation (1) the expected value of *P* can be calculated if we know the expected value of the diversity statistics *F*_*ISM*_ and *F*_*SMM*_.

5 $$ E\left[P\right]=1-\frac{E\left[{F}_{ISM}\right]}{E\left[{F}_{SMM}\right]} $$

Where:

6 $$ E\left[{F}_{SMM}\right]={\sum}_{x=1}^tE\left[{F}_{SMM}|{T}_{ij}=x\right]P\left({T}_{ij}=x\right) $$

7 $$ E\left[{F}_{ISM}\right]={\sum}_{x=1}^tE\left[{F}_{ISM}|{T}_{ij}=x\right]P\left({T}_{ij}=x\right) $$

The same approach was also used to obtain the expected values of *MSH* and *DH*. The analytical equations to calculate those expected values are explained in the Appendix.

### Simulations to analyze changes in homoplasy measures

We used a simulation framework to test the accuracy of our estimates for the summary statistics *π*, *F* and *F* ^*i*^ under the *ISM* and the *SMM* along with our estimates for the homoplasy values *P*, *MSH* and *DH*. We used our modified version of the coalescent simulator *msHOT* [21, 22] to generate two different sets of haplotypes (*hSMM* and *hISM*) for each simulated genealogy. The modified version of *msHOT* is available at https://github.com/dortegadelv/HomoplasyMetrics. *msHOT* was used to make simulations under the stepwise demographic expansion model, where we used a value of *θ*_1_ = 30, *θ*_0_ = 0.03 and ten different values of *τ* {1.5, 3, 4.5, 6, 7.5, 9, 10.5, 12, 13.5, 15}. For each value of *τ*, we performed 100 simulations of 150 haplotypes with 6 linked *SSRs*. The ten command lines used for those simulations are shown in the Appendix (Command Line 1).

We also did 100 simulations for 9 different numbers of linked *SSRs* in the haplotype, going from *L* = 2 to *L* = 10 to examine how changes in the value of *L* affect *P*, *MSH* and *DH*. We simulated 150 haplotypes in each simulation, where the values of the demographic parameters were set to *θ*_1_ = 5*L*, *θ*_0_ = 0.005*L*, *τ* = *L* = 2*tuL*, where we kept the parameters *t* and *u* fixed to a certain value such that 2*tu* = 1. Notice that the divergence time *t* is kept fixed regardless of the number of linked *SSRs L* in these simulations. The nine command lines used for these simulations are shown in the Appendix (Command Line 2).

### Underestimation of expansion time

We quantified the underestimation of expansion time due to homoplasy using a metric called *TS*, which we define as

8 $$ TS=\frac{\widehat{\tau_{ISM}}-\widehat{\tau_{SMM}}}{\widehat{\tau_{ISM}}} $$

Values of the estimated expansion time τ for haplotypes *hISM* and *hSMM*, $\widehat{\tau_{ISM}}$ and $\widehat{\tau_{SMM}}$ respectively, were obtained using the method by Schneider and Excoffier (1999), implemented in the software *Arlequin* [24]. This method infers the parameters *θ*_0_, *θ*_1_ and *τ* based on the observed distribution of pairwise differences between haplotypes, also called mismatch distribution, and its expectation under a stepwise demographic expansion model [25]. This approach assumes that there is no homoplasy in the sample of haplotypes, therefore any differences between $\widehat{\tau_{ISM}}$ and $\widehat{\tau_{SMM}}$ are due to homoplasious mutations present in *hSMM*. Following [15], to use *Arlequin* for the *hSMM* analysis we coded the *SSRs* as binary data, where the number of repeats were coded with ‘1’ and shorter alleles were coded filling the difference in repeats with ‘0’ .

We simulated 100 replicates of 150 haplotypes with 6 linked *SSRs* for each of 10 different values of τ {1.5, 3, 4.5, 6, 7.5, 9, 10.5, 12, 13.5, 15}. We set a value of *θ*_1_ equal to 30 and 60, which has the same order of magnitude of the value of *θ*_1_ estimated for the *Pinus caribaea* dataset [26] employed in this study *(see Pinus caribaea* dataset*)*, and a value of *θ*_0_ which was 1000 smaller than *θ*_1_ for all simulations. The command lines used for the simulations are shown in the Appendix. Command Line 3 and 4 were used for the simulations done where *θ*_1_ = 30 and *θ*_1_ = 60, respectively.

The value of each homoplasy measure and *TS* was computed for each replicate of each simulation and the relationship of each homoplasy measure with *TS* was analyzed. In the simulations done with a value of *θ*_1_ = 30, we removed 1 out of the 1000 simulations we performed where that simulation was the only that had a *TS* value smaller than -10 (see Additional file 1: Figure S1 for details on the removed simulation).

### Estimation of homoplasy and expansion time using ABC

We used another modified version of the program *msHOT* [21, 22], also available at https://github.com/dortegadelv/HomoplasyMetrics, to implement an *ABC* algorithm that estimates the posterior distribution of demographic parameters *θ*_0_, *θ*_1_ and *τ* and the posterior predictive distribution of the three measures of homoplasy *DH*, *MSH* and *P* (see Appendix for details about the implementation of the *ABC* algorithm). We employed three summary statistics previously used [27] to estimate demographic parameters in a model of population growth: The mean of the variance in the size of the *SSRs* across loci (*V*), the expected heterozygosity averaged across loci (*H*) and the number of distinct haplotypes (*a*). We used the mode of the posterior distribution and posterior predictive distribution as point estimates of the demographic parameters and homoplasy measures, respectively (see Appendix and Additional file 1: Figure S2 for a discussion on why we employed the mode as a point estimate). We also quantified the relative bias and estimated the 50%, 75% and 90% coverage of the demographic parameters and homoplasy measures to ascertain the quality of the point estimates and the inferred posterior distributions (see Appendix). The relative bias is the average difference between the estimated and true value of the parameter divided by its true value [28] . The 50%, 75% and 90% coverage are the proportion of times that the true value is within the 50%, 75% and 90% credible interval.

We compared the real value of the homoplasy measures *P*, *MSH* and *DH* against the estimated values of the homoplasy measures using our *ABC* approach in 100 simulations of 150 haplotypes with 6 linked *SSRs* with parameters *θ*_1_ = 30 and *θ*_0_ = 0.03 and 10 different values of τ {1.5, 3, 4.5, 6, 7.5, 9, 10.5, 12, 13.5, 15}, where 10 simulations were done for each τ value. The ten command lines used for the simulations are shown in the Appendix (Command Line 5). We also estimated the three homoplasy measures in the simulations we explain in the next paragraph.

We compared the performance of three different methods to infer *τ* and *θ*_1_ in three different sets of 100 simulations of 150 haplotypes with 6 linked *SSRs* done using three different *τ* values {3,6,9}, a *θ*_1_ = 30 and a *θ*_0_ = 0.03. The three command lines for these simulations are shown in the Appendix (Command Line 6). One of the three methods we used to estimate *τ* and *θ*_1_ is our *ABC* approach, and the other two methods use the mismatch distribution to estimate those demographic parameters: 1) One of those methods is the approach taken by [3] as implemented in the software *Arlequin* [24], which assumes that there is no homoplasy in the data (Least Squares approach without taking Homoplasy into account, *LSWH*). 2) The other method we used is a maximum-pseudolikelihood estimator that uses a model where it is assumed that homoplasy can occur in the data [16] (Maximum Pseudolikelihood using a model with Homoplasy, *MPH*). Code for that method was kindly provided by Miguel Navascués.

### Estimation of homoplasy and population expansion times in a *Pinus caribaea* dataset

We used a dataset of 7 *SSR* loci from 88 individuals of the species *Pinus caribaea* to estimate *τ* along with the homoplasy measures *MSH* and *DH*. This dataset is a subset of the data previously published in [26], where an analysis of population structure from four species of *Pinus* subsection *Australes*, including *Pinus caribaea*, identified four different groups (groups I-IV). We took the group containing the largest number of individuals distributed in Central America (group II), and retained only the individuals sampled from Central America in that group (88 out of 93 individuals) for our analysis. A hypothesis of population expansion could not be rejected using information from the mismatch distribution in group II [26], making this group suitable for analysis of expansion. We used *ABC*, *LSWH* and *MPH* to estimate *τ* in that dataset. The estimations of $\widehat{\tau}$ in the three methods used above were later transformed to years using a mutation rate of 5.5 *X* 10^−5^ per *SSR* per generation [29] and a generation time of 42.5 years [26].

We also report the 95% confidence interval of the estimation of $\widehat{\tau}$ with *LSWH*, *MPH* and *ABC* using a parametric bootstrap approach as in *Arlequin* [24]*.* We report the 95% confidence intervals for the *ABC* method instead of the 95% credible intervals to compare the 95% confidence intervals created with *ABC* with those obtained using *LSWH* and *MPH*. For each particular inference method (*LSWH*, *MPH* or *ABC),* the approach involves the simulation of 1000 datasets of 88 individuals with 7 *SSR* using the demographic parameters estimated for the *Pinus caribaea* data using a particular inference method. The value of the parameter *τ* ^·^ from each of the 1000 datasets was estimated using the inference method under study. Then, for a confidence level of *α* = 0.05, the approximate limits of the confidence interval were defined as the *α*/2 and 1 – *α*/2 percentile values of the 1000 values of *τ* ^·^. This parametric bootstrap approach was also used to estimate the 95% confidence interval of *MSH* and *DH* using the *ABC* method and 1000 simulations done using the demographic parameters inferred by *ABC*.

## Results

### Response of different measures of homoplasy to differences in expansion time

**Fig. 1:**
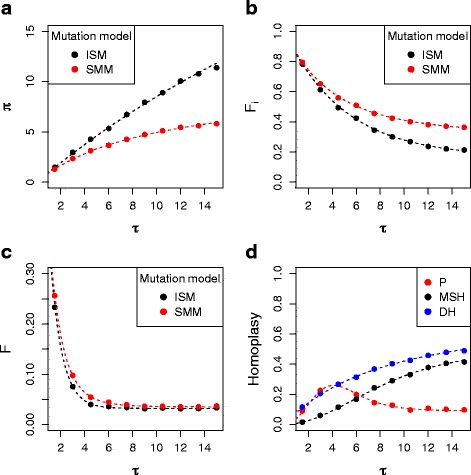
Homoplasy values in the stepwise demographic expansion model for different values of the expansion time parameter *τ*. The points in each plot are the average values for each statistic across 100 simulations for plots (**a**-**c**), those average values were used to calculate the mean values of the homoplasy index (*P*), mean size homoplasy (*MSH*) and distance homoplasy (*DH*) that are plotted as points in (**d**). The dashed lines are the approximated expected values estimated from our derivations. **a** *π*_*ISM*_ and *π*_*SMM*_; **b** ${F}_{ISM}^i$ and ${F}_{SMM}^i$; **c** *F*_*ISM*_ and *F*_*SMM*_ **d** *P*, *MSH* and *DH*

First we evaluated the response in the three different measures of homoplasy and their components, *π*, *F* ^*i*^ and *F,* to changes in expansion time under the demographic stepwise expansion model in haplotypes containing completely linked *SSRs*. We thereby corroborate our theoretical expectations for the different metrics and found that our simulations validate their predictions (Fig. 1a-d).

As can be seen in Fig. 1a-b, the accumulation of homoplasious mutations causes a monotonic increase in the difference between ${F}_{ISM}^i$ and ${F}_{SMM}^i$ and between *π*_*SMM*_ and *π*_*ISM*_ with the expansion time, something that is not observed for the difference between the two measures of haplotype homozygosity *F*_*ISM*_ and *F*_*SMM*_ (Fig. 1c). This translates to both *MSH* and *DH* increasing steadily with expansion time, while *P* has a parabolic relationship (Fig. 1d) and stays at a constant value close to 0.09 when τ is equal or larger than 8 (Fig. 1d). Therefore, the values of *P* do not seem likely to relate to underestimation of population expansion time, in contrast with *MSH* and *DH*. Additionally, we found that the number of linked *SSRs* in the haplotype does not influence the values of *MSH* and *DH*, but it does change the value of *P* given a fixed divergence time *t* (Additional file 1: Figure S3).

### The relation between different measures of homoplasy and underestimation of τ

**Fig. 2:**
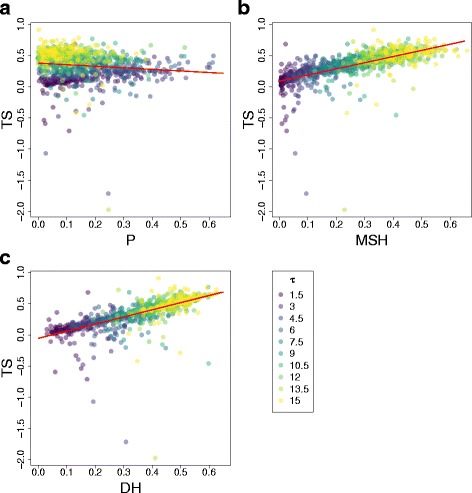
Linear relationship between *TS* and three measures of homoplasy: **a** *P* (ρ = −0.1282, intercept = 0.3783, slope = −0.2509, *p*-value = 4.83e^−5^), **b** *MSH* (ρ =0.6903, intercept = 0.0893, slope = 0.9868, *p*-value <2.2e^−16^) and **c** *DH* (ρ =0.6989, intercept = −0.0541, slope = 1.1424, *p*-value <2.2e^−16^) in 999 simulations made with the demographic parameters *θ*_0_ = 0.03, *θ*_1_ = 30 and 10 different values of *τ*

Simulations of demographic expansion under different values of τ reveal that the standard homoplasy index *P* is not strongly correlated with *TS*, which measures the underestimation of τ due to homoplasy (Fig. 2a, Pearson’s *ρ* = −0.1282, *p*-value = 4.8*10^−5^). Contrarily, *MSH* and *DH*,have a stronger correlation with an underestimation of τ, where *MSH* has a slightly lower correlation with *TS* (Fig. 2b, *ρ* = 0.6903, *p*-value <2.2*10^−16^) than *DH*, which has the strongest correlation with *TS* of all the homoplasy measures inspected (Fig. 2c, *ρ* = 0.6989, *p*-value <2.2*10^−16^). Correlation between the latter two measures was strong (0.9208), whereas neither of them was strongly correlated with *P* (*ρ* between *MSH* and *P* = −0.2685; *ρ* between *DH* and *P* = −0.0977). Simulations with a higher value of *θ*_1_ = 60 **(**Additional file 1: Figure S4), produced a nearly identical relationship between *DH*, *MSH* and *TS* (*ρ* between P and *TS* = −0.2617; *ρ* between *MSH* and *TS* = 0.8673; *ρ* between *DH* and *TS* = 0.8777), showing that *DH* and *MSH* are robust predictors of an underestimation of τ while *P* is not.

### ABC estimates of homoplasy and expansion time

**Fig. 3:**
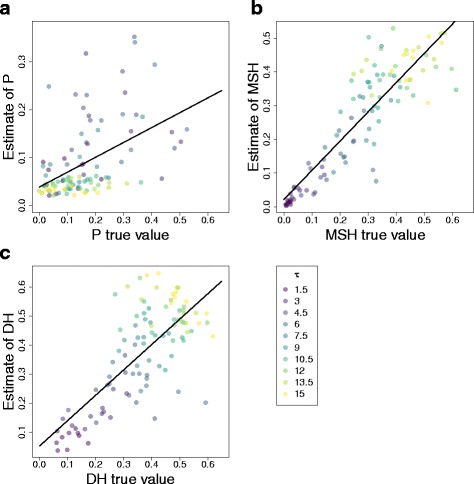
*ABC* estimates of **a** *P*, **b** *MSH* and **c** *DH* compared with their true values in 100 simulations done with the demographic parameters *θ*_0_ = 0.03, *θ*_1_ = 30 and 10 different values of *τ*. A linear model was fitted to analyze the relationship between each homoplasy measure true value and their *ABC* estimate of *P* (ρ = 0.4864, intercept = 0.0385, slope = 0.3104, *p*-value = 2.88e^−7^), *MSH* (ρ = 0.8809, intercept = 0.0220, slope = 0.8667, *p*-value <2.2e^−16^) and *DH* (ρ = 0.7399, intercept = 0.0512, slope = 0.8771, *p*-value <2.2e^−16^)

We used simulated data to evaluate the estimation of homoplasy metrics on an *ABC* framework. We performed linear regressions of the estimated values of the homoplasy measures obtained by our *ABC* approach on their true homoplasy values (Fig. 3) We also measured the relative bias and the correlation between the estimated and true values of the homoplasy measures. On simulations done over a range of τ values, we found that our estimates of *MSH* and *DH* were highly correlated with their real values (*r* = 0.881 and *r =* 0.740, respectively) and their relative bias was small (relative bias = −0.040 and 0.030, respectively), indicating that *MSH* and *DH* values are well estimated by our *ABC* approach. On the other hand, the estimates of *P* had a smaller correlation with their true values (*r* = 0.486) and their relative bias is −0.132, indicating that our *ABC* approach underestimates *P* values by approximately 13.2%. The underestimation can also be seen in Fig. 3. Despite differences in the quality of the point estimates of *P*, *MSH* and *DH*, we found that the 50%, 75% and 95% coverage of the homoplasy measures indicate that the inferred posterior distribution of those measures are well estimated (Additional file 1: Table S1 and Appendix).

We performed more simulations to analyze the performance of the *ABC* approach on simulations done using the same demographic parameters. We evaluated this by creating three sets of simulations done over a single value of τ (τ = 3, 6 or 9). We found that the 50%, 75% and 90% coverage indicate that the posterior distributions of the homoplasy measures are correctly inferred (Additional file 1: Table S2 and Appendix). Second, we found that the average relative bias was small for all homoplasy measures (relative bias = 0.053, 0.043 and 0.068 for *P*, *MSH* and *DH*, respectively; Additional file 1: Table S3). This indicates that, on average, the *ABC* method slightly overestimates the value of the homoplasy measures by approximately 5% on these sets of simulations.

**Fig. 4:**
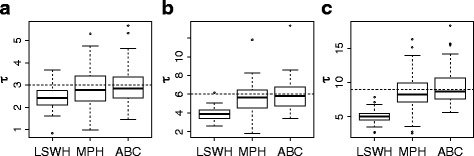
Estimation of *τ* using three methods (*LSWH*, *MPH* and *ABC*). The boxplots of the estimation of *τ* were done on simulations where *θ*_1_ = 30, *θ*_0_ = 0.03 and three different values of *τ* were used, **a** *τ* = 3, **b** *τ* = 6 and **c** *τ* = 9. 100 simulations were performed for each value of *τ*. The actual value of *τ* in each plot is displayed with the dashed line

Apart from estimating homoplasy measures, we also used *ABC* to estimate the value of τ. We found that *ABC* and the pseudo-likelihood estimator (*MPH*) perform equally well to obtain estimates of the value of τ, showing that both methods can correct for the effects of homoplasy. As expected, the expansion time is strongly underestimated by the method that does not take homoplasy into account (*LSWH*), particularly for older expansion events where there are higher values of *MSH* and *DH* (Fig. 4). Additionally, we found that *ABC* gave good estimations of the value of *θ*_1_, compared to *LSWH* and *MPH* which gave overestimations of the actual value of *θ*_1_ (Additional file 1: Figure S5), in line with previous studies done using *LSWH* [3] and *MPH* [16]. It must be pointed out that in *ABC*, as in any Bayesian method, the estimates of the parameters depend on the prior distributions used for the parameters. Prior distributions should contain all the possible demographic parameter values [30] and should not be very wide to avoid low acceptance rates in the *ABC* algorithm (step 5 of the *ABC* algorithm in the Appendix).

### Population expansions and homoplasy in data of *Pinus caribaea* populations from central America

The time of expansion for one population of *Pinus caribaea* in Central America was obtained using *LSWH*, *MPH* and *ABC* (Table 1). We found that the only method where homoplasy is not taken into account, *LSWH*, produces lower estimates of $\widehat{\tau}$ compared to *ABC* and *MPH*, suggesting that homoplasy may cause the expansion time to be underestimated by approximately 100,000 years in this case. The 95% confidence intervals of $\widehat{\tau}$ for all methods is large however (Table 1). *ABC*-based estimates of homoplasy are 0.11 and 0.246 for *MSH* and *DH* respectively, which agrees with the theoretical estimates of 0.106 and 0.269 obtained for those homoplasy measures using equations (24, Appendix) and (17, Appendix) given the demographic parameters estimated using *ABC*.

**Table 1:** Estimates of homoplasy and times of expansion in the population of *Pinus caribaea* analyzed

$\widehat{\tau_{LSWH}}$	$\widehat{\tau_{MPH}}$	$\widehat{\tau_{ABC}}$	MSH	DH	Theoretical estimate of MSH	Theoretical estimate of DH
4.074 (1.359 – 6.086)	5.994 (1.732 – 10.297)	5.591 (2.645 – 11.005)	0.115 (0.026 – 0.244)	0.246 (0.106 – 0.415)	0.106	0.269
Time of expansion in years (*LSWH*)			Time of expansion in years (*MPH*)		Time of expansion in years (*ABC*)	
224,900 (75,000 – 335,900)			330,800 (95,600 – 568,400)		308,600 (146,000 – 607,400)	

The estimated values of the time of expansion were obtained using three different methods (*LSWH*, *MPH* and *ABC*). The estimated values of *MSH* and *DH* were obtained using *ABC*. The numbers inside the parentheses denote the upper and lower limits of the 95% confidence interval for the parameter or homoplasy measure. The theoretical estimates of *MSH* and *DH* were estimated using the values of *τ* and *θ*_1_ obtained using *ABC* and the Eqs. (24) and (17)

## Discussion and conclusions

Here we propose a homoplasy metric, *DH*, which measures the proportion of pairwise differences that are not observed due to homoplasy. Our theoretical estimates and simulations confirm that the mean number of pairwise differences not counted due to homoplasy increase when population expansion times are older, causing a monotonic increase in the value of *DH* (Fig. 1). We also confirm that *DH* has a strong, linear relationship with underestimation of population expansion times using classical methods of inference based on pairwise differences (Fig. 2), with older expansion times leading to the expected higher *TS* [16] and corresponding increase in *DH*. This in contrast to the standard homoplasy index for multi-locus haplotypes, *P*, which shows no clear relation to *TS* and, starting from a certain τ value, actually decreases as a function of expansion time. The latter can be clearly understood from the expected relation between expansion time and homozygosity under the *ISM* and *SSM* model. This shows the value of a homoplasy metric that directly captures the underestimation of the number of mutations [18] when trying to capture effects on demographic inference.

Although conceptually and empirically *DH* most closely relates to the way that homoplasy causes underestimation of expansion time, the average decrease of per-locus heterozygosity, *MSH*, has a rather similar relation to population expansion time (Fig. 1) and also correlates strongly with the underestimation of the population expansion time (Fig. 2). It has been shown that in constant population sizes the value of *MSH* is determined by *θ* [12], the expected number of mutations between a pair of sequences, so the fact that it also increases with *τ* is not entirely surprising. Our theoretical estimates indeed confirm that *MSH* increases monotonically with expansion time under the stepwise demographic expansion model (Fig. 1) and also show that there is a relationship between the number of mutations, predicted by older coalescent times due to older population expansions, and *MSH* on the stepwise population expansion model. Additionally, *MSH* and *DH* are not affected by changes in the number of linked *SSRs* analyzed, while *P* does depend on the number of linked *SSRs* studied.

The usefulness of homoplasy metrics such as *DH* and *MSH* depends in part on how well they can be estimated from data. We have shown that we can obtain reasonable average estimates of *MSH* and *DH* using *ABC* [31]. Compared to *MASH*, the *ABC* method we propose is not biased by the fraction of homoplasy unmeasurable by *MASH*. It thereby offers a natural solution to the quantification of homoplasy. Additionally, *ABC* can estimate the posterior distribution of demographic parameters of interest through explicit modeling of *SSR* evolution under different demographic scenarios. The approach proved successful in correcting for bias in the inference of expansion time, with similar performance to *MPH* [16] which also explicitly accounts for homoplasy assuming the *SSR’s* evolve according to a *SMM*. One advantage of *ABC*, in addition to allowing for direct estimates of homoplasy, is that more complicated mutational models of *SSR* evolution can easily be incorporated. This could be important, as many *SSR* are known not to evolve in a strictly stepwise manner [32].

Given the potential for erroneous demographic inference when using linked SSR, it is important to obtain such homoplasy estimates from empirical data. With our *ABC* approach, we were able to estimate values of *MSH* and *DH* in a published dataset of *Pinus caribaea*. We found that the underestimation of the expansion time assuming a model that does not take homoplasy into account is of around 80,000 to 100,000 years, a reduction of around 28 to 32% compared to the value estimated with methods that use a more realistic model of *SSR* evolution where homoplasious events are possible. As with the *ABC* approach proposed here, other authors have suggested to use model-based approaches to infer past demographic events using linked *cpSSR* markers in spruces [11]. Since *ABC* simulation based approaches provide an estimate of homoplasy, we believe that *ABC* approaches are useful to quantify the effect of homoplasy on demographic parameters and summary statistics of interest. To our knowledge, this is the first time that homoplasy parameters have been inferred using population genetic data.

## Additional file

Additional file 1: Figures S1-S5 and **Tables S1-S3**. (DOCX 3133 kb)

## Appendix

### Expected values of the parameters π, F^i^ and F given a certain coalescent time

Given a certain coalescent time *T*_*ij*_ between lineages *i* and *j*, it is possible to estimate the expected values of a diversity statistic *λ*, such as the mean number of differences between two haplotypes (*π*)*,* the expected homozygosities at one *i* SSR locus (*F* ^*i*^) and the expected homozygosity in a linked *SSR* multilocus haplotype (*F*) under the *ISM* and the *SMM*. If we can estimate that quantity, we can use this equation:

9 $$ E\left[\lambda \right]={\sum}_{x=1}^tE\left[\lambda |{T}_{ij}=x\right]P\left({T}_{ij}=x\right) $$

to obtain the expected value of those diversity statistics given different parameters of a stepwise demographic expansion model. Using those expected values, we can calculate estimates of the homoplasy measures *DH, MSH* and *P*.

We will start by defining the values of the summary statistics *π*_*ISM*_ and *π*_*SMM*_ given a particular coalescent time. *π*_*ISM*_ and *π*_*SMM*_ define the value of *π* in a set of linked multilocus *SSR* haplotypes *hISM* evolving under the infinite sites model *ISM*, and in a set of linked multilocus *SSR* haplotypes *hSMM* that evolved under the symmetrical stepwise mutation model *SMM*, respectively. The expected value of *π*_*ISM*_ for *L* loci given a certain coalescent time *T*_*ij*_ and a mutation rate per generation in one loci equal to *u* is:

10 $$ E\left[{\pi}_{ISM}\right|\ {T}_{ij}\ \Big]=\mathrm{Y}=2 Lu{T}_{ij} $$

To obtain the expected value of *π*_*SMM*_, we used the random walk model formulas from [33] to estimate the expected number of differences between a pair of *SSR’s* given that *x* mutations have taken place in the two lineages since their divergence from a common ancestor:

11 $$ If\ x\  is\  odd:E\Big[{\pi}_{SMM}\ \left|\# mutations=x\right]=\frac{1}{2^{x-1}}\frac{x+1}{2}\left(\begin{array}{c}x\\ {}\frac{x+1}{2}\end{array}\right) $$

12 $$ If\ x\  is even:E\Big[{\pi}_{SMM}\ \left|\# mutations=x\right]=\frac{1}{2^{x-2}}\frac{x}{2}\left(\begin{array}{c}x-1\\ {}\frac{x}{2}\end{array}\right) $$

The number of mutations *x* that happened between each pair of *SSRs* is distributed as a Poisson random variable with mean$\mathrm{Y}=2 Lu{T}_{ij}$. Therefore, the probability of having an *x* number of mutations is:

13 $$ P\left[\# mutations=x\right]=\frac{{\left(\frac{\mathrm{Y}}{L}\right)}^x{e}^{-\frac{\mathrm{Y}}{L}}}{x!} $$

Where *L* is the number of loci. Using those facts, we can obtain the expected number of differences *π*_*SMM*_ using the following formula:

14 $$ E\left[{\pi}_{SMM}|\ {T}_{ij}\right]=L{\sum}_{x=0}^{\infty }E\Big[{\pi}_{SMM}\ \left|\# mutations=x\right]P\left[\# mutations=x\right] $$

To be practical, the past sum was carried out until $\sum_{x=0}^rP\left[\# mutations=x\right]$ was bigger or equal to 0.999 for the smallest possible value of r.

Following (9), (10), and (14), we can obtain the expected values of *π*_*SMM*_ and *π*_*ISM*_ using:

15 $$ E\left[{\pi}_{ISM}\right]={\sum}_{x=1}^tE\left[{\pi}_{ISM}|{T}_{ij}=x\right]P\left({T}_{ij}=x\right) $$

16 $$ E\left[{\pi}_{SMM}\right]={\sum}_{x=1}^tE\left[{\pi}_{SMM}|{T}_{ij}=x\right]P\left({T}_{ij}=x\right) $$

The values of *E*[*π*_*SMM*_] and *E*[*π*_*ISM*_] can be combined to obtain the expected value for the homoplasy parameter *DH*:

17 $$ E\left[ DH\right]=\frac{E\left[{\pi}_{ISM}\right]-E\left[{\pi}_{SMM}\right]\ }{E\left[{\pi}_{ISM}\right]} $$

We can also obtain the expected value of *MSH* by estimating the expected values of the homozygosity parameters for each of the *i SSR* loci under the infinite sites model ${F}_{ISM}^i=1-{H}_{ISM}^i$ and the stepwise mutation model ${F}_{SMM}^i=1-{H}_{SMM}^i$. If we have *L* SSR’s:

18 $$ E\left[{F}_{ISM}^i|\ {T}_{ij}\right]={\left(1-u\right)}^{2{T}_{ij}}={e}^{-2{T}_{ij}u}={e}^{-\mathrm{Y}/L} $$

To obtain the value of ${F}_{SMM}^i$ we followed [34] derivations of the expected values of heterozygosity. They defined the probability of having an equal number *x* of mutations that increase or decrease the number of repeats in a *SSR* given that 2x mutations have occurred:

19 $$ P\left[\ x\  mutations that increase repeat number\ |\# mutations=2x\right]=\left(\begin{array}{c}2x\\ {}x\end{array}\right){\left(\frac{1}{2}\right)}^{2x} $$

And given that the probability of having 2x mutations is:

20 $$ P\left[\# mutations=2x\right]=\frac{{\left(\frac{\mathrm{Y}}{L}\right)}^{2x}{e}^{-\frac{\mathrm{Y}}{L}}}{(2x)!} $$

Then, summing over all possible values of x, we obtain the value of *F*_*SMM*_:

21 $$ E\left[{F}_{SMM}^i|\ {T}_{ij}\right]={\sum}_{x=0}^{\infty }P\left[\ x\  mutations that increase repeat number\ |\# mutations=2x\right]P\left[\# mutations=2x\right] $$

This sum was also done until $\sum_{x=0}^rP\left[\# mutations=x\right]$ was bigger or equal to 0.999 for the smallest possible value of r. Using (9) we get:

22 $$ E\left[{F}_{SMM}^i\right]={\sum}_{x=1}^tE\left[{F}_{SMM}^i|{T}_{ij}=x\right]P\left({T}_{ij}=x\right) $$

23 $$ E\left[{F}_{ISM}^i\right]={\sum}_{x=1}^tE\left[{F}_{ISM}^i|{T}_{ij}=x\right]P\left({T}_{ij}=x\right) $$

Combining those equations, an analytical equation for the expected value of *MSH* can be obtained:

24 $$ E\left[ MSH\right]=1-\frac{\sum_{i=1}^L\frac{E\left[{F}_{ISM}^i\right]}{E\left[{F}_{SMM}^i\right]}}{L}=1-\frac{E\left[{F}_{ISM}^i\right]}{E\left[{F}_{SMM}^i\right]} $$

We can also derive a theoretical estimate of homozygosity in the haplotype composed of the *L SSR* loci under the *ISM* and *SMM*. Under the *ISM*, the expected homozygosity value is equal to:

25 $$ E\left[{F}_{ISM}|\ {T}_{ij}\right]={\left(1- Lu\right)}^{2\ {T}_{ij}}={e}^{-2L\ {T}_{ij}u}={e}^{-\mathrm{Y}} $$

To calculate the expected homozygosity under the *SMM* in a haplotype containing a set of linked *SSR’s*, we use the following formula:

26 $$ E\left[{F}_{SMM}|\ {T}_{ij}\right]={\sum}_{x=0}^{\infty }P\left[ two\  haplotypes\  are\  identical\  by\  state\right|\# mutations=x\Big]P\left[\# mutations=x\right] $$

To calculate the probability that two haplotypes are identical by state given that x mutations happened before they coalesce to a common ancestor it is necessary to: 1) Count all the possible ways in which *x* mutations could happen to make the two haplotypes identical by state and 2) Divide that number between all the possible ways in which *x* mutations could be distributed along the haplotypes. Many of the possible distributions of mutations that could make two haplotypes identical by state have a very low probability of occurring, where most of those cases involve having a very high number of mutations, therefore ignoring those cases does not alter much the value of *E*[*F*_*SMM*_] calculated. We found that the following formula provides a good approximation to the value of *F*_*SMM*_:

$$ E\left[{F}_{SMM}|\ {T}_{ij}\right]\approx P\left[\# mutations=0\right]+L\ {\sum}_{x=1}^LP\Big[2x\  mutations landed\  on\  the same microsatellite\  and\  two\  identic al $$

$$ by\  state haplotypes were produced\left]+{\sum}_{x=2}^L\ \left(\begin{array}{c}L\\ {}x\end{array}\right)P\right[2\  mutations landed $$

$$ on\ x\  different microsatellites\  and\  two\  identical\  by\  state haplotypes $$

$$ were\ p roduced\Big] $$

The past formula can also be expressed as:

27 $$ E\left[{F}_{SMM}|\ {T}_{ij}\right]\approx P\left[\# mutations=0\right]+L\ {\sum}_{x=1}^L\ P\left[2x\  mutation s landed\  on\  the same microsatellite\ |\# mutations=2x\right]P\left[\# mutations=2x\right]\ P\left[x\  mutation s that increase repeat number\ \right|\# mutations=2x\Big]+{\sum}_{x=2}^L\ \left(\begin{array}{c}L\\ {}x\end{array}\right)P\left[2\  mutation s landed\  on\ x\  different microsatellites\ |\# mutations=2x\right]P\left[\# mutations=2x\right]\prod_{i=1}^xP\left[1\  mutation that increase repeat number\ |\# mutations=2\right] $$

Where *P*[#*mutations* = 2*x*] is given by (20), substituting Υ/L by Υ *P*[*x mutations that increase repeat number* | # *mutations* = 2*x*] is given by (19) and:

28 $$ P\left[2x\  mutations landed\  on\  the same microsatellite\ |\# mutations=2x\right]={\left(\frac{1}{L}\right)}^{2x} $$

And, using the multinomial distribution, we can get:

29 $$ P\left[2\  mutations landed\  on\ x\  different microsatellites\ \right|\# mutations=2x\Big]=\frac{2x!}{\prod_{i=1}^x2!}{\left(\frac{1}{L}\right)}^{2x} $$

Then, using (9), we get:

30 $$ E\left[{F}_{SMM}\right]={\sum}_{x=1}^tE\left[{F}_{SMM}|{T}_{ij}=x\right]P\left({T}_{ij}=x\right) $$

31 $$ E\left[{F}_{ISM}\right]={\sum}_{x=1}^tE\left[{F}_{ISM}|{T}_{ij}=x\right]P\left({T}_{ij}=x\right) $$

Using those results, we can compute an approximate expectation for the value of *P*, which estimates the haplotypic reduction in heterozygosity due to homoplasy, as:

32 $$ E\left[P\right]=1-\frac{E\left[{F}_{ISM}\right]}{E\left[{F}_{SMM}\right]} $$

### ABC algorithm

The input of the algorithm is the value of three summary statistics *S* calculated from a set of linked haplotypes with *L* SSR loci. Those three statistics *S* are *V*, *H* and *a*. We define *V* as the mean of the variance in the size of the *SSRs* across loci, *H* is the expected heterozygosity averaged across loci and *a* is the number of distinct haplotypes. The output of this *ABC* algorithm is a sample of the posterior distribution of the demographic parameters *θ*_0_, *θ*_1_, *τ* and a sample of the posterior predictive distribution of the homoplasy measures *P*, *MSH* and *DH* [35]*.* The *ABC* algorithm uses the following steps:

1. Read a set of *H* haplotypes with *L* linked *SSRs* per haplotype and compute their values of *V*, *H* and *a*.
2. Simulate values of *θ*_0_, *θ*_1_ and *τ* from their respective prior distributions.
3. Simulate a number of *H* haplotypes with *L* linked SSRs with a genealogy created under the coalescent model employing the demographic stepwise expansion model and the above values of *θ*_0_, *θ*_1_ and *τ*.
4. Estimate *V**, *H**, *a*, P, MSH, DH* from the simulated linked *SSRs*.
5. If all of |*V-V**|/*V*, |*H-H**|/*H* and |*a-a**|/*a* are less than *ε* = 0.01, then record the values of *θ*_0_, *θ*_1_, *τ*, *P*, *MSH* and *DH*.
6. Return to 2) until *N* values of *θ*_0_, *θ*_1_, *τ*, *P*, *MSH* and *DH* are obtained (which means that we have obtained *N* accepted simulations).

The prior distribution used for *θ*_1_ was uniform (0,200). For the reduction in the value of *θ*_1_ to *θ*_0_ we used a prior distribution that followed a uniform(0, 0.01), based on that reduction we obtained the value of *θ*_0_ for each simulation. The prior distribution for *τ* was dependent on the value $\overset{\sim }{\theta_1}$ sampled from each simulation and was distributed as uniform(0, 2*$\overset{\sim }{\theta_1}$). That prior distribution for *τ* was motivated by the fact that in a constant population size with a *θ* value of X, the expected coalescent time multiplied by 2Lu is 2X if the number of samples is large. Therefore, we decided to leave the expected coalescent time in a constant population multiplied by 2Lu as an upper bound for the prior distribution of *τ*. The same set of prior distributions was used for all the *ABC* analysis reported in this paper.

The *N* values of *θ*_0_, *θ*_1_, *τ* are samples from the distribution *P*(Θ| | *V* − *V**| /*V* < *ε*,  | *H* − *H**| /*H* < *ε*, | *a* − *a**| /*a* < *ε*) of those parameters if we define Θ as one demographic parameter. On the other hand,the *N* values of *P*, *MSH* and *DH* recorded are samples from the posterior predictive distribution *P*(H| | *V* − *V**| /*V* < *ε*,  | *H* − *H**| /*H* < *ε*, | *a* − *a**| /*a* < *ε*), where H is a homoplasy measure [35]. The importance of choosing an appropriate *ε* value has been particularly well explained in a recent review [36], where the authors highlight that choosing a small *ε* value is important to make sure that the distributions *P*(Θ| | *V* − *V**| /*V* < *ε*,  | *H* − *H**| /*H* < *ε*, | *a* − *a**| /*a* < *ε*) and *P*(H| | *V* − *V**| /*V* < *ε*,  | *H* − *H**| /*H* < *ε*, | *a* − *a**| /*a* < *ε*) converge to the posterior distribution *P*(Θ| S) and *P*(H| S), respectively. In our case, we used an *ε* = 0.1, a value previously used in analysis containing the same summary statistics used here [27]. We evaluated if the *ABC* approach, including the choice of that value of *ε*, gave accurate point estimates of Θ and H and distributions that converge to the posterior distributions of Θ and H in the next section.

We used the function ‘density’ from R to build an estimate of the posterior distributions of each of the three parameters and the three homoplasy measures in the model based on the N values recorded of the homoplasy measures and the demographic parameters. The modes of the posterior and posterior predictive distributions were used as estimates of each of the parameters and homoplasy measures, respectively. We chose to use the mode, instead of the median or the mean, as our point estimate of the parameter and homoplasy measures for reasons that will be detailed in the next section.

To test the exactitude of our *ABC* method to estimate demographic parameters and homoplasy measures, we created 3 sets of simulations, where each simulation contained a set of 150 haplotypes composed of 6 linked *SSRs*. One set was used to analyze the change in the estimation of the homoplasy measures at ten different values of *τ* {1.5, 3, 4.5, 6, 7.5, 9, 10.5, 12, 13.5, 15}, performing 10 simulations for each value of *τ*. The other three sets were used to analyze the estimation of the homoplasy measures in three specific values of *τ* {3,6,9}, where we performed 100 simulations for each value of *τ*. Our *ABC* approach was run in each replicate of each simulation of those four sets until *N*= 10,000 simulated acceptances were obtained. Due to computational constraints associated with the calculation of the confidence intervals, we used N=1,000 simulated acceptances in the *Pinus caribaea* dataset.

### Quality of demographic parameter estimates

We used a set of metrics to measure the quality of our parameter estimates using *ABC*, as recommended in [30]. One of those metrics is the relative bias, which is the average difference between the estimated and true value of the parameter divided by its true value [28]. First, we compared the relative bias when using the median, mode and mean as point estimates of the demographic parameters *τ* and *θ*_1_, and of the homoplasy measures *P*, *MSH* and *DH*. This comparison was performed in the three different sets of 100 simulations described in the past section where the values of *τ* were 3, 6 and 9. We found that, on average, using the mode of the posterior distribution as a point estimate provided a smaller relative bias for estimates of *τ*, *MSH, P* and *DH* (Additional file 1: Table S3). The only parameter where using the mean of the posterior distribution provided a better point estimate was *θ*_1_ (Additional file 1: Table S3). For simplicity and consistency, we decided to use the mode of the posterior distribution for all the point estimates we report. This decision is motivated by the fact that we were more interested in estimating *τ* and the homoplasy measures *P*, *MSH* and *DH*.

We evaluated our inferred posterior distributions estimating the 50%, 75% and 90% coverage, defined as the fraction of times that the true parameter estimate is inside the 50%, 75% and 90% credible intervals [28]. We define the X% credible interval as the X% highest posterior density interval (HPD). If the credible intervals created using the inferred posterior distributions are correct and have good coverage properties, the true parameter value should be present inside the 50%, 75% and 90% credible intervals with probabilities of 50%, 75% and 90%, respectively. We found that, on average, the 50%, 75% and 90% credible intervals for the demographic parameter *τ*, and the homoplasy measures *P*, *DH* and *MSH* had good coverage properties. *τ* and the homoplasy measures had a probability of being inside the credible interval that matched expectations, since the absolute difference between those probabilities and the expected probabilities was smaller than 0.05 (Additional file 1: Table S2). In the case of the parameter *θ*_1_, we found that, on average, the parameter had slightly more conservative and broad intervals, since the true parameter tended to be inside the credible interval more often than what was expected, with the difference between the expected and observed probabilities of *θ*_1_ being in the credible interval was between 0 and 0.1 (Additional file 1: Table S2). This indicates that our *ABC* method, including our selection of prior distributions and *ε*, is inferring accurate posterior distributions for the homoplasy measures and *τ*, and slightly biased posterior distributions for the parameter *θ*_1_.

### Command lines

- Command Line 1:
  ./msHOT 150 100 -t 30 -eN $tau 0.001 -Q -z 6 -seeds 1 2 5
  Where $tau took ten different values {0.025, 0.05, 0.075, 0.1, 0.125, 0.15, 0.175, 0.2, 0.225, 0.25}.
- Command Line 2:
  ./msHOT 150 100 -t $Theta -eN 0.1 0.001 -Q -z $i -seeds 1 2 $i
  Where $i took values going from 2 to 9, and $Theta was equal to $i times 5.
- Command Line 3:
  ./msHOT 150 100 -t 30 -eN $tau 0.001 -Q -z 6 -seeds 1 2 3
  Where $tau took ten different values {0.025, 0.05, 0.075, 0.1, 0.125, 0.15, 0.175, 0.2, 0.225, 0.25}.
- Command Line 4:
  ./msHOT 150 200 -t 60 -eN $tau 0.001 -Q -z 6 -seeds 1 2 3
  Where $tau = {0.0125, 0.025, 0.0375, 0.05, 0.0625, 0.075, 0.0875, 0.1, 0.1125, 0.125}
- Command Line 5:
  ./msHOT 150 10 -t 30 -eN $tau 0.001 -Q -z 6 -seeds 1 2 3
  Where $tau took ten different values {0.025, 0.05, 0.075, 0.1, 0.125, 0.15, 0.175, 0.2, 0.225, 0.25}.
- Command Line 6:
  ./msHOT 150 100 -t 30 -eN $tau 0.001 -Q -z 6 -seeds 2 3 4
  Where $tau = {0.05, 0.1, 0.15}.

## Abbreviations

ABC: Approximate Bayesian Computation
cpSSR: Chloroplast simple sequence repeats
DH: Distance Homoplasy
GBS: Genotyping-by-sequencing
HPD: Highest posterior density interval
ISM: Infinite Sites Model
LSWH: Least Squares approach without taking Homoplasy into account
MASH: Molecular size Homoplasy
MPH: Maximum pseudolikelihood using a model with Homoplasy
MSH: Mean size Homoplasy
P: Homoplasy index
RADseq: Restriction site-associated DNA sequencing
SMM: Stepwise mutation model
SSR: Simple sequence repeats
