# Supplementary material for: Appropriate homoplasy metrics in linked SSRs to predict an underestimation of demographic expansion times
Source: BMC Evol Biol. 2017 Sep 11;17:213. doi: 10.1186/s12862-017-1046-4 (PMC5594565; doi:10.1186/s12862-017-1046-4)
Supplement: Additional file 1: Figures S1-S5 — and Tables S1-S3. (DOCX 3133 kb) [file 12862_2017_1046_MOESM1_ESM.docx]

**Additional files**

**Figure S1**.- A) Distribution of pairwise differences in 100 simulations done under an infinite sites model with demographic parameters ($\tau=15, \theta_{1}=30, \theta_{0}=0.03$). We obtained a simulation with a *TS* value equal to -301.913 out of the 100 simulations done with the parameters $\tau=15, \theta_{1}=30, \theta_{0}=0.03$. The inferred value of $\tau$ for that simulation in the *hSMM* was equal to 6.967 and it was equal to 0.023 in the *hISM*, therefore *TS* = (6.967 – 0.023)/ 0.023 = 301.913. The low inferred value of $\tau$ in the *hISM* is the reason behind the high value of *TS*, since *TS* is affected by very low values estimated in *hISM*. We analyzed the reason behind the low inferred value of of $\tau$ in the *hISM* by looking at the distribution of pairwise differences in that particular simulation. We compared the shape of the distribution of pairwise differences of two random simulations done with a $\tau$ value equal to 15 (*Random sim 1, Random sim 2*), the simulation with a *TS* value is equal to -301.913 (*Multimodal sim*), and the average distribution of pairwise distances for 100 simulations we made (*All sims average*). Compared to the other three distributions, *Multimodal sim* was multimodal. Under a population expansion, the distribution of pairwise distances is expected to be unimodal (Rogers and Harpending 1992). The least squares approach of Schneider and Excoffier (1999) finds the parameters of a population expansion that minimize the difference between the observed distribution of pairwise differences and a unimodal distribution of pairwise differences from a population under expansion. Schneider and Excoffier (1999)’s method found that the best unimodal distribution that fits the multimodal distribution of *Mutimodal sim* has a low $\tau$ value. *Multimodal sim* is the only simulation we discarded in our analysis presented in Figure 2.

B) Inferred values of $\tau$ in *hISM* ($\tau=15, \theta_{1}=30, \theta_{0}=0.03$, 100 sims). The actual value of $\tau$ in *hISM* is indicated by the dashed line while the median is indicated by the middle line in the boxplot (14.832). We found that the estimated values of $\tau$ in *hISM* are accurate, indicating that the low inferred value of $\tau$ in *Multimodal sim* is uncommon. After the inferred value of $\tau$ in *Multimodal sim* (0.023), the next lowest inferred value of $\tau$ is 3.266.

C-E) Linear relationship between *TS* and three measures of homoplasy: a) *P (*Intercept: 0.421; slope: -2.544; r: -0.031; p-value: 0.334*)*, b) *MSH (*Intercept: -0.244; slope: 1.1093; r: 0.018; p-value: 0.565*)* and c) *DH* (Intercept: 0.293; slope: -0.738; r: -0.011; p-value: 0.738) in 1000 simulations made with the demographic parameters $\theta_{0}$ = 0.03, $\theta_{1}$ = 30 and 10 different values of $\tau$. Contrast this with Figure 2, where we did not include the *Multimodal sim*. The inclusion of *Multimodal sim* causes the values of r to go closer to 0 and the p-values to become bigger than 0.05. We decided to drop *Multimodal sim* from the main analysis presented in Figure 2 since we were interested in the average trends in the reduction of estimated $\tau$ values due to homoplasy using *TS*, and we did not want to be biased by one uncommon simulation.

 **Figure S2**.- Point estimates of $\tau$ and $\theta_{1}$ using the median, mean and mode of their respective posterior distributions. The boxplots of the estimation of $\tau$ ( A), C) and E) ) and $\theta_{1}$ ( B), D) and F) ) were done on 100 simulations where $\theta_{1}$ = 30, $\theta_{0}$ = 0.03 and three different values of $\tau$ were used: $\tau$ = 3 for A) and B); $\tau$ = 6 for C) and D); $\tau$ = 9 for E) and F). We display the actual value of the demographic parameter analyzed, $\theta_{1}$ or $\tau$, in each plot with a dashed line.

**Figure S3.-** Homoplasy values in the stepwise demographic expansion model for different numbers of linked SSRs in the haplotype given a fixed divergence time *t*. The points in each plot are the average values for each statistic across 100 simulations for plots A)-C), those average values were used to calculate the mean values of *P*, *MSH* and *DH* that are plotted as points in D). The dashed lines are the approximated expected values estimated from our derivations. A) $\pi_{ISM}$ and $\pi_{SMM}$; B) $F_{ISM}^{i}$ and $F_{SMM}^{i}$; C) $F_{ISM}$ and $F_{SMM}$ D) *P*, *MSH* and *DH*.

**Figure S4.-** Linear relationship between *TS* and three measures of homoplasy: a) *P* (ρ = -0.2617, intercept = 0.4137, slope = -0.3412, p-value < 2.2e^-16^), b) *MSH* (ρ = 0.8673, intercept = 0.1109, slope = 0.7829, p-value < 2.2e^-16^) and c) *DH* (ρ = 0.8777, intercept = -0.0558, slope = 1.1309, p-value < 2.2e^-16^) in 1000 simulations made with the demographic parameters $\theta_{0}$ = 0.06, $\theta_{1}$ = 60 and 10 different values of $\tau$.

**Figure S5**.- Estimation of $\theta_{1}$ using three methods (*LSWH*, *MPH* and *ABC*). The boxplots of the estimation of $\theta_{1}$ were done on 100 simulations where $\theta_{1}$ = 30, $\theta_{0}$ = 0.03 and three different values of $\tau$ were used: A) $\tau$ = 3, B), $\tau$ = 6 and C) $\tau$ = 9. The actual value of $\theta_{1}$ in each plot is displayed with the dashed line.

**Table S1**.- 50%, 75% and 90% coverage of the homoplasy measures *P*, *MSH* and *DH* over 100 simulations done on a range of $\tau$ values {1.5, 3, 4.5, 6, 7.5, 9, 10.5, 12, 13.5, 15}

| Homoplasy measures | 50% Coverage | 75% Coverage | 90% Coverage |
| --- | --- | --- | --- |
| *P* | 0.52 | 0.76 | 0.90 |
| *MSH* | 0.52 | 0.78 | 0.92 |
| *DH* | 0.52 | 0.75 | 0.88 |

**Table S2**. 50%, 75% and 90% coverage of the demographic parameters $\tau$ and $\theta_{1}$, and the homoplasy measures *P*, *MSH* and *DH.*

| Relative bias on $\tau$ estimates | | | |
| --- | --- | --- | --- |
| Real $\tau$ value | 50% Coverage | 75% Coverage | 90% Coverage |
| 3 | 0.52 | 0.73 | 0.91 |
| 6 | 0.52 | 0.77 | 0.94 |
| 9 | 0.53 | 0.76 | 0.9 |
| Average across $\tau$ ={3, 6, 9} values | 0.52 | 0.75 | 0.92 |
| Relative bias on $\theta_{1}$ estimates | | | |
| Real $\tau$ value | 50% Coverage | 75% Coverage | 90% Coverage |
| 3 | 0.67 | 0.86 | 0.97 |
| 6 | 0.55 | 0.81 | 0.95 |
| 9 | 0.5 | 0.79 | 0.94 |
| Average across $\tau$ ={3, 6, 9} values | 0.57 | 0.82 | 0.95 |
| Relative bias on *P* estimates | | | |
| Real $\tau$ value | 50% Coverage | 75% Coverage | 90% Coverage |
| 3 | 0.61 | 0.78 | 0.9 |
| 6 | 0.55 | 0.83 | 0.89 |
| 9 | 0.48 | 0.77 | 0.92 |
| Average across $\tau$ ={3, 6} values | 0.55 | 0.79 | 0.90 |
| Relative bias on *MSH* estimates | | | |
| Real $\tau$ value | 50% Coverage | 75% Coverage | 90% Coverage |
| 3 | 0.56 | 0.79 | 0.9 |
| 6 | 0.49 | 0.83 | 0.94 |
| 9 | 0.51 | 0.75 | 0.92 |
| Average across $\tau$ ={3, 6, 9} values | 0.52 | 0.79 | 0.92 |
| Relative bias on *DH* estimates | | | |
| Real $\tau$ value | 50% Coverage | 75% Coverage | 90% Coverage |
| 3 | 0.52 | 0.75 | 0.91 |
| 6 | 0.57 | 0.8 | 0.93 |
| 9 | 0.51 | 0.69 | 0.85 |
| Average across $\tau$ ={3, 6, 9} values | 0.53 | 0.75 | 0.90 |

**Table S3**. Relative bias using the mean, median and mode as point estimates of the demographic parameters $\tau$ and $\theta_{1}$, and the homoplasy measures *P*, *MSH* and *DH.*

| Relative bias on $\tau$ estimates | | | |
| --- | --- | --- | --- |
| Real $\tau$ value | Mode | Mean | Median |
| 3 | -0.024 | 0.136 | -0.146 |
| 6 | -0.019 | 0.349 | -0.085 |
| 9 | 0.041 | 0.666 | 0.009 |
| Average across $\tau$ ={3, 6, 9} values | -0.001 | 0.384 | -0.074 |
| Relative bias on $\theta_{1}$ estimates | | | |
| Real $\tau$ value | Mode | Mean | Median |
| 3 | -0.902 | -0.886 | -0.915 |
| 6 | -0.804 | -0.730 | -0.817 |
| 9 | -0.688 | -0.500 | -0.697 |
| Average across $\tau$ ={3, 6, 9} values | -0.798 | -0.705 | -0.810 |
| Relative bias on *P* estimates | | | |
| Real $\tau$ value | Mode | Mean | Median |
| 3 | -0.117 | 0.251 | 0.160 |
| 6 | 0.016 | 0.403 | 0.304 |
| 9 | 0.261* | 1.575* | 1.199* |
| Average across $\tau$ ={3, 6} values | 0.053 | 0.743 | 0.554 |
| Relative bias on *MSH* estimates | | | |
| Real $\tau$ value | Mode | Mean | Median |
| 3 | -0.003 | 0.353 | 0.211 |
| 6 | 0.015 | 0.178 | 0.122 |
| 9 | 0.117 | 0.176 | 0.158 |
| Average across $\tau$ ={3, 6, 9} values | 0.043 | 0.236 | 0.164 |
| Relative bias on *DH* estimates | | | |
| Real $\tau$ value | Mode | Mean | Median |
| 3 | -0.014 | 0.214 | 0.131 |
| 6 | 0.051 | 0.156 | 0.120 |
| 9 | 0.168 | 0.216 | 0.201 |
| Average across $\tau$ ={3, 6, 9} values | 0.068 | 0.195 | 0.151 |

*We ignored one simulation where the true value of *P* was equal to 0 when we estimated the relative bias of P on the simulations where the real value of $\tau$ was equal to 9.
